# Nature prescription for patients with chronic respiratory diseases^☆^

**DOI:** 10.1016/j.waojou.2026.101351

**Published:** 2026-02-27

**Authors:** Marilyn Urrutia-Pereira, Dirceu Solé, Tonya Winders, Verónica Riquelme Martinez, Rovana Kinas Bueno, Debora da Cruz Payão Pellegrini, Jeferson Rafael Bueno, Felipe Haniel Derré Torres, Camila Girotto Alberti, Bruna Simoneto Marques, Barbara Angelo de Moraes, Ana Clara Sevá, Daniel Barba Kaestner, Herberto Jose Chong-Neto, Tari Haahtela

**Affiliations:** a Federal University of Pampa, Uruguaiana, Brazil; b Brazilian Association of Allergy and Immunology, São Paulo, Brazil; c Federal University of São Paulo, São Paulo, Brazil; d Instituto Pensi, São Paulo, Brazil; e GAAPP, Global Allergy and Airways Patient Platform, Vienna, Austria; f Hospital de Clinicas, Federal University of Paraná, Curitiba, Brazil; g Skin and Allergy Hospital, Helsinki University Hospital, University of Helsinki, Helsinki, Finland

**Keywords:** Nature prescription, Nature deficiency, Chronic obstructive pulmonary disease, Asthma, Green spaces, Respiratory health, Mindfulness

## Abstract

Nature prescription is a relatively new clinical approach consisting of recommendations of exposure to natural environments as a complementary intervention to support health. Especially in the treatment of asthma and chronic obstructive pulmonary disease (COPD), increased connection to wider nature appears useful. Nature prescription integrates green space practices such as outdoor walks, other low-grade exercise, contemplation, and mindfulness. We have prepared a scoping review of the relevant literature on nature prescription for patients with chronic respiratory diseases, considering population studies but also clinical experience. The results indicate that regular exposure to green space contributes to increased physical activity, reduced stress and anxiety, improved lung function, reduced need for rescue medication, and better adherence to treatment. A protocol is also proposed to evaluate these outcomes. We conclude that integrating the use of green space and natural outdoor elements into multidisciplinary care represents an accessible, safe enough, and potentially effective strategy to promote respiratory health.

## Introduction

Recent evaluations of the global burden of disease indicate that much of the leading causes of death and years of chronic ill health are preventable and associated with the deterioration and pollution of ecosystems.^1^

Chronic respiratory diseases, such as asthma and chronic obstructive pulmonary disease (COPD), represent a serious public health burden, affecting millions of people worldwide and generating high social and economic costs. These conditions are characterized by chronic and often persistent symptoms, such as dyspnea, cough, and functional limitations, as well as recurrent hospitalizations impacting patients quality of life.^2^, ^3^, ^4^

The negative impact of climate change and biodiversity/nature loss on human health has also elicited recognition of various positive environmental, natural factors protecting health.^5^

In addition to the pharmacological treatment recommended by guidelines for asthma^3^ and COPD,^4^ the search for complementary and integrative approaches has led healthcare professionals to explore non-pharmacological interventions, such as nature prescriptions.^6^, ^7^, ^8^ This practice recommends regular contact with natural environments to promote physical and mental well-being.^9^, ^10^, ^11^

In addition to fostering physical activity, green spaces offer sensory stimulation, stress reduction, and increased body and environmental awareness. A wealth of scientific evidence has shown that exposure to natural environment can benefit the respiratory system and emotional state and is particularly promising in combating chronic diseases.^12^

According to the "Biodiversity Hypothesis", reduction in environmental and dietary biodiversity to which an individual is exposed compromises the richness and diversity of the human microbiota and increases the risk of allergies and other inflammatory, non-communicable diseases.^13^, ^14^, ^15^, ^16^

Adequate environmental microbial exposure can be achieved by restoring contact to urban green space with natural richness and microbial phylogenetic diversity,^17^^,^^18^ bringing natural elements back into our everyday lives to breathe, eat, drink, and touch.^19^

This article presents a scoping review of the clinical and physiological potential of nature prescription in people with chronic respiratory diseases, discussing its applicability, and limitations. It also proposes a health care protocol based on nature prescription for adults, elderly, and children with chronic respiratory diseases.

## Literature search

The literature review was conducted based on searches of international scientific databases, such as PubMed, Scopus, Web of Science, and ScienceDirect, using combinations of the descriptors: “nature prescription”, “green space”, “respiratory diseases”, “asthma”, “COPD”, “NDVI”, “urban nature”, and “health outcomes”. Studies published between 2009 and 2025 were included primarily, with an emphasis on systematic reviews, observational trials, and meta-analyses that addressed the relationship between exposure to natural environments and respiratory outcomes in populations with chronic diseases.

## Nature prescription

Nature prescription is a recent approach for patients with chronic diseases recommending regular exposure to natural environments as part of clinical care and follow-up. Nature-based activities are incorporated into health management plans for people of all ages, but especially for those with chronic diseases.^5^

Nature-based prescribing involves healthcare professionals recommending their patients to spend time in nature, whether through written prescription, verbal counseling, or referral to another provider.^20^, ^21^

Spending time in nature provides several benefits, including social and environmental connection^22^ (Table 1).

**Table 1:** Evidence-based benefits of contact with nature^5^^,^^12^

Change	Result	Consequence
Physical	Improvement of cardiovascular, respiratory and motor functions	Decreased blood pressure, improved lung function (FEV1, forced expiratory volume in 1 s), exercise tolerance
Autonomic regulation	Balance between sympathetic and parasympathetic systems	Increased heart rate variability, deep relaxation
Mental/Emotional	Reduction of anxiety and depression	Decreased cortisol, increased serotonin and dopamine, improved mood
Psychosocial	Strengthening self-esteem, social bonds and a sense of belonging	Increased empathy, motivation and connection with groups
Cognitive	Increased focus, attention and mental clarity	Increased performance in cognitive tasks, decreased mental rumination
Behavioral	Increased adherence to self-care and physical activity	Reduction of sedentary behavior, improvement of circadian rhythm
Sleep/Recovery	Improved sleep quality and physical and emotional recovery	Reduced insomnia, improved quality of REM (rapid eye movement) sleep, and less fatigue
Spiritual	Expanding the meaning of life, gratitude and acceptance	Reduction of spiritual suffering and strengthening of inner faith

## Prescribing nature to patients with chronic respiratory diseases

Contact with nature promotes several physiological and environmental effects that can directly benefit the respiratory system, especially in patients with chronic respiratory diseases. These effects work synergistically, contributing to both symptom relief and slowing down disease progression.

### Improved air quality

Natural environments, such as urban forests and parks, have significantly lower concentrations of air pollutants such as fine particulate matter (PM2.5), nitrogen dioxide (NO_2_), and tropospheric ozone (O_3_), compared to densely populated urban areas. Vegetation acts as a biological filter, absorbing pollutants and emitting oxygen, resulting in cleaner, less irritating air for inflamed airways.^8^^,^^23^

### Reducing inflammation and activating immune modulation

Studies indicate that volatile organic compounds emitted by plants, such as phytoncides (antimicrobial essential oils found in trees such as pine and cedar), have anti-inflammatory and immunoregulatory effects. Inhaling these compounds can reduce lung inflammation and modulate the immune system, particularly benefiting patients with asthma and COPD.^24^^,^^25^

### Enriching human microbiome

Chronic noncommunicable diseases share common characteristics: microbial dysbiosis, immune dysfunction, and a tendency toward an inflammatory response.

The lung microbiota plays a fundamental role in immune homeostasis and protection against respiratory pathogens.^26^ Evidence suggests that biodiversity interventions have the potential to diversify the human microbiota in a short period of time.^27^ Exposure to green spaces diversifies the gut and skin microbiota and alters their composition toward healthier human microbiota profiles.^28^ This may improve immunoregulatory pathways and provide an incentive for future prophylactic approaches to reduce the risk of immune-mediated diseases in urban societies.^29^

### Stimulating breathing and ventilation

By inducing relaxation and reducing anxiety, the natural environment facilitates the adoption of slower, deeper breathing patterns, which improve alveolar ventilation and tissue oxygenation. Furthermore, light activities such as hiking stimulate lung capacity and exercise tolerance, elements often compromised in patients with respiratory diseases.^26^^,^^27^

### Reduction of oxidative stress

Urban environmental stress, including noise, pollution, and confinement, promotes the production of free radicals that aggravate chronic inflammatory diseases, including lung diseases. The natural environment, by reducing psychophysiological stress, contributes to the reduction of oxidative stress, an important factor in the progression of COPD and bronchial remodeling in patients with asthma.^12^

### Psychological and behavioral effects

Disorders such as anxiety and depression are common in patients with chronic respiratory disease and often worsen physical symptoms. Nature acts as a mood regulator, decreasing activation of the hypothalamic-pituitary-adrenal (HPA) axis, resulting in reduced cortisol level and the perception of dyspnea^10^^,^^30^, ^31^, ^32^, ^33^.

### Treatment adherence and quality of life

By encouraging patient autonomy, nature-based prescriptions promote active engagement in self-care. The simple practice of walking in a park, in addition to improving physical health, contributes to self-esteem and a sense of well-being, which can promote adherence to conventional medical treatment.^12^

### Other measurable effects

Direct health-related outcomes include: a) reduction in asthma exacerbations and COPD symptoms;^25^^,^^34^ b) reduction in blood pressure;^35^^,^^36^ c) reduction in smoking and alcohol consumption;^37^ d) reduction in the use of anxiolytics and antidepressants;^37^ and e) improvement in sleep.^38^

## Mechanisms

The human body is its own ecosystem and is connected to the ecosystems of wider nature through eating (drinking), touching, and breathing. The epigenetically guided crosstalk between the ecosystems is mainly mediated by microbes and biogenic chemicals (BVOCs) driving the immune regulation.^6^

Biodiverse environment challenges the innate immunity and boosts tolerance. For example, early exposure to a microbe-rich farming environment protects children from asthma and allergy by shaping the innate immunity response.^39^

The mental and sociocultural factors also matter. The addition of natural elements, such as creating parks and green space, using potted plants and even offering views to green environment through windows have measurable effects on health and well-being.^40^

**Fig. 1:**
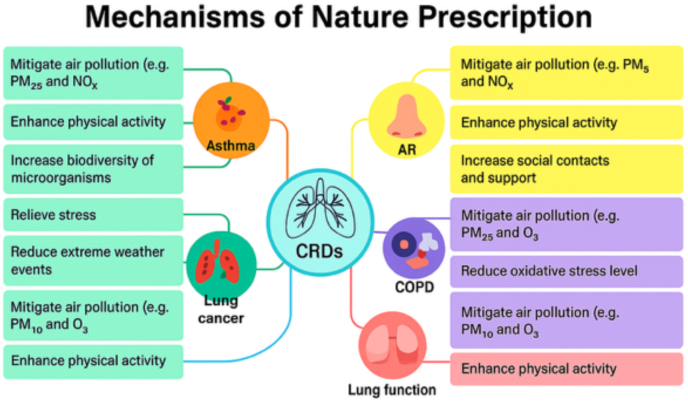
Beneficial factors involved in the prescription of nature for chronic respiratory diseases (AR = Allergic Rhinitis, CRD = Chronic Respiratory Diseases, COPD = Chronic Obstructive Pulmonary Diseases)

The more gross scale beneficial factors of nature prescription in various respiratory conditions are illustrated in Fig. 1.

## Main barriers to prescribe for nature^22^^,^^41^^,^^42^

Despite evidence that prescribing for nature is effective in treating patients with chronic respiratory diseases, some barriers can hinder its implementation (Table 2).

**Table 2:** Barriers that interfere with the prescription of nature

Structural barriers	a) Lack of accessible green spaces in urban areas b) Inadequate infrastructure (safety, accessibility, signage, transportation) c) Extreme weather (intense cold, heat, pollution)
Institutional barriers	a) Lack of formal guidelines in clinical protocols and health systems b) Lack of integration between the health, environmental, and urban planning sectors c) Low public interest and investment in nature-based health programs
Professional barriers	a) Lack of knowledge or skepticism on the part of physicians and health teams regarding the benefits and scientific evidence b) Lack of specific training in environmental health c) Limited consultation time in everyday practice for preventive or alternative approaches
Sociocultural barriers	a) Fear, insecurity, or stigma associated with public spaces in certain social groups b) Patients with low adherence to non-pharmacological strategies or without a habit of outdoor leisure activities c) Linguistic, cultural, or physical barriers (disability, reduced mobility)
Economic barriers	a) Transportation costs to nature areas b) Lack of personnel or other resources for organized therapeutic projects (guided trails, green therapies) c) Inequalities in access to nature between social classes

## Nature prescription program for patients with chronic respiratory diseases

*Objectives for successful implementation:*

• Reducing barriers to contact with nature, such as cost, access, lack of time, and the ability to connect participants with the environment.
• Feasibility with cost-effective implementation, using existing resources to support future communications and evaluations.
• Creating a consortium with partners from various sectors willing and able to support a nature prescription program.
• Raising awareness of the role of nature both for physical and mental health promotion.
• Provide information that can be evaluated to identify barriers to participation and make further recommendations^12^^,^^43^

### Measuring environmental greenness and grayness

The first step is to measure the “green” and/or “gray” environmental exposure of the region where the protocol will be implemented. Green characteristics generally refer to the amount of vegetation in an area. Gray characteristics include building density, imperviousness, or road network connectivity.^44^

*a) Normalized Difference Vegetation Index (NDVI* ***)***

The NDVI is a satellite-based index used to measure the density and health of vegetation in an area. It is calculated based on the difference between near-infrared (NIR) and visible red (RED) light reflected by plants. [NDVI = NIR - RED/NIR + RED. NIR = Near-Infrared, healthy vegetation reflects a lot. RED = healthy vegetation absorbs a lot].^45^

NDVI is important because it allows us to assess:^40^ a) the amount of green space around a person's residence, b) the relationship between urban vegetation and respiratory, mental, or cardiovascular health, c) environmental changes that impact public health policies, and d) areas with higher NDVI can be linked to lower mortality, lower incidence of asthma, and lower stress.

NDVI has limitations that can affect its accuracy and usefulness a) because the values do not differentiate the reflectance between trees and grass, even with different volumes of vegetation and different levels of ecosystem functionality, and b) they cannot differentiate between allergenic and non-allergenic species or wild and cultivated pastures^46^^,^^47^

b) *Land Cover Classification – CORINE Land Cover*

The Coordination of Information on the Environment Land Cover System (CORINE CLC) is a standardized European method for classifying and monitoring land use and land cover. Its hierarchical categories are widely used in environmental, urban, and public health studies, including NDVI exposure assessments.^48^

Identifying areas from an Environmental Health perspective is important because it allows: a) classifying urban versus green areas for population exposure; b) relating green areas to physical and mental health indicators; c) assessing the impact of urban density on respiratory and cardiovascular diseases.

c) *Residential Surrounding Grayness (RSG)*

RSG is an indicator that measures the proportion of urbanized, impervious, and built areas (such as concrete, asphalt, roofs, and roads) surrounding an individual's residence.^8^^,^^49^

This measurement is important for health: a) a high proportion of RSG is associated with greater air pollution, less contact with nature, increased stress, and increased temperature (heat island); b) it can negatively impact chronic respiratory diseases, cardiovascular health, and mental health; c) it is an important counterpoint to the NDVI, which measures surrounding vegetation; and d) it analyzes the balance between areas green (NDVI) and gray (RSG) soils to improve urban planning for health.

## Suggestion for a nature prescription protocol

Selected patients (over 6 years of age) must sign the informed consent form and the assent form (for those under 18 years of age). Children must have the form completed by their parents or guardians.

a) *Clinical history*

The following data must be obtained: respiratory diagnosis (asthma, COPD), frequency and severity of symptoms (dyspnea, cough), history of recent hospitalizations or exacerbations, current use of medications (bronchodilators, corticosteroids, anxiolytics, antidepressants, antihypertensives, cardiovascular medications), comorbidities (allergic rhinitis, diabetes, obesity, heart disease, bone problems), current level of physical activity and lifestyle habits (diet, alcohol and tobacco consumption, sleep).

b) *Physical examination*

It will consist of lung auscultation, measurement of respiratory and heart rates, obtaining body mass index, measurement of systemic blood pressure, and if possible, measuring O2 saturation at rest and performing a 6-min walk test.

c) *Additional tests*

Depending on the available options, patients may undergo the following tests, depending on their age group: spirometry before and after inhaled bronchodilator use, pulse oximetry at rest and after minimal exertion, electrocardiogram (ECG) at rest (especially for those over 50 or with cardiovascular comorbidities), laboratory tests (complete blood count, blood glucose, glycated hemoglobin, total cholesterol and fractions, triglycerides), thyroid function tests, liver and kidney function tests, chest X-ray (if not performed in the last 12 months), assessment of possible immediate hypersensitivity (total and/or specific IgE or skin prick test), Asthma Control Test (ACT, for those over 12 years of age),^50^ Childhood Asthma Control Test (CACT), for children 4–12 years of age,^51^ and COPD Assessment Test (CAT).^52^

Participants may also be assessed by 1 or more of the instruments in Table 3.

**Table 3:** Instruments used in the initial assessment of patients

The Hogg Eco-Anxiety Scale (HEAS)^53^	Measures anxiety related to environmental issues such as climate change and environmental degradation.
Stress perception scale (EPS10) (EPS10)^54^	Assesses the perception of stress
Hospital anxiety and depression scale (HADS)^55^	Detects mild degrees of anxiety and depression.
Positive and negative affect scale (PANAS)^56^	Assesses the frequency and intensity with which people experience affective states.
Connection with nature scale (ECN)^57^	Assesses the affective aspect of the person-environment relationship.
Insomnia severity index (IGI)^58^	Assesses the severity of insomnia and its outcomes.
Generic quality of life questionnaire SF-36^59^	Assesses quality of life.
Baecke questionnaire (physical activity)^60^	Assesses physical activity in the last 12 months using 3 scores (occupational physical activity, leisure-time physical exercise, leisure-time physical activity and mobility).

### Monitoring the nature prescription at patient or group level

During the interventions the patients can be monitored by a multidisciplinary team comprised of physicians, other healthcare professionals, medical students, occupational therapists, psychologists, physical therapists, or other available professionals who wish to participate.

After each intervention, discussions can be held about coping and adjusting the “dose” of nature, based on the response of each patient/group.

*The main objectives of the prescription of nature are:*

• Improving respiratory function and blood pressure.
• Stimulating conscious breathing.
• Connecting the ecosystem of the human body to the wider ecosystems.
• Enriching the biodiversity of the human microbiome.^61^
• Reducing cortisol and markers of anxiety and stress.
• Reducing sympathetic nervous system hyperactivity (relaxation).
• Expanding sensory perception and a sense of vitality.
• Inducing states of mindfulness and meditation.
• Improving mood and sleep quality.

## Options for interventions

### Walking, hiking

The main activities proposed during the trail include warm-up, silent walking, and sensory pauses (smelling, observing, lying down, and contemplating sounds), close eyes, and perform abdominal breathing, observing the air entering you and returning to the environment.^12^^,^^62^

Other interventions may include walking, running in parks, cycling, gardening, or farming, or simply "being" in green environments (*shinrin-yoku*, or forest bathing),^53^, ^54^, ^55^, ^56^, ^57^, ^58^, ^59^, ^60^, ^61^, ^62^, ^63^, ^64^, ^65^, ^66^, ^67^ strolling, hugging trees, and listening to birdsong. However, it is important to consider that some trees produce allergenic pollen that can trigger conditions such as allergic rhinitis or allergic asthma, making it essential to consider their allergenic potential when choosing the regions where nature activities will be carried out and the time of year.^68^

### Other exercise

The integration of body movement like dance, mindfulness, yoga with nature is a powerful way to care for physical, emotional, and spiritual health.

The clinical objectives are:^69^, ^70^, ^71^, ^72^, ^73^, ^74^, ^75^, ^76^

• Physical and respiratory rehabilitation.
• Reducing stress and anxiety.
• Promoting self-esteem and creative expression.
• Connection with the body and the environment.
• Widening scale of motion and body awareness.
• Improving balance and motor coordination.
• Strengthening the bond with nature as a therapeutic space.

Sessions will respect the physical limits of participants and include free-flowing dance walks, intuitive dance with headphones, spontaneous choreography inspired by the landscape, circle dancing, or group dancing, always in a safe and pleasant environment.^77^, ^78^, ^79^

a) *Employing blue space*

Exposure to blue spaces has been associated with better health outcomes, particularly regarding chronic stress and burnout, anxiety, insomnia, and muscle tension, mild to moderate depression, chronic fatigue, promoting preventative mental health, contributing to improvements in physical and respiratory health.^80^, ^81^, ^82^, ^83^, ^84^, ^85^, ^86^

“Blue” natural therapy includes contemplative walks by the water's edge, fishing, auditory meditation with the sound of water, partial immersion (feet or hands in the water), mindful floating or supervised sea bathing, and reflective writing after aquatic contact (excursions, fishing). Activities should be performed during quiet times, in safe places, always respecting the patient's physical limits.

b) *Mindfulness*

The term mindfulness refers to the trait or mental state of being intentionally attentive to the present experience with acceptance, openness, kindness, and non-judgment.^87^^,^^88^

Mindfulness and compassion practices are fundamentally important strategies for strengthening the cognitive and emotional foundations of connection, both promote resilience commitment to more conscious and assertive choices in the face of the climate crisis.

The therapeutic objectives are*:*^89^, ^90^, ^91^

• Reducing mental hyperactivity.
• Promoting emotional regulation and reducing cortisol.
• Strengthening presence and body awareness.
• Stimulating focus, mental clarity, and internal stability.
• Creating a conscious connection with the natural environment.
• Improving sleep quality and mood.
• Increasing emotional resilience.
• Improving respiratory health
• Promoting psychological and emotional resilience

The location for practicing mindfulness should be quiet, safe, with vegetation or a natural view. The practice can be performed individually or in a group, guided by a facilitator properly trained in the adopted protocol.

c) *Yoga*

Yoga is an ancient practice that comprises a set of psychophysical techniques aimed at balancing body, mind, and breath. In patients with chronic respiratory diseases, yoga has been shown to be effective in improving lung function, reducing anxiety, inflammatory markers related to asthma and COPD, and improving quality of life, especially when associated with interventions in natural environments. 92, 93, 94, 95, 96, 97, 98

The therapeutic objectives are:^92^, ^93^, ^94^, ^95^, ^96^, ^97^, ^98^

• Promote the integration of breathing, movement, and attention.
• Reduce sympathetic activity (stress) and promote deep relaxation.
• Improve mobility, balance, and coordination.
• Strengthen the connection with the natural environment as a healing space.
• Stimulate self-knowledge and self-regulation.

The choice of location should favor silence, shade, and stable ground.

d) *Spirituality*

This type of intervention provides the patient with the awareness that they are connected to something beyond their individual self, but which can be grounded in a more balanced existence.

The therapeutic objectives are:^99^, ^100^, ^101^, ^102^

• Helping patients in search of meaning or inner reconnection.
• Relieving existential stress and spiritual suffering.
• Processing deeper self-care and reconnection with values.
• Relieving chronic illnesses with emotional impact.
• Strengthening the bond with something greater (transcendence).
• Stimulating a sense of connection with life and nature.
• Reducing symptoms of helplessness, emptiness, and existential isolation.
• Reducing anxiety, stress, and depression.
• Improving treatment adherence.

Suggested activities include: a) contemplative walk, b) gratitude ritual at sunrise or sunset, c) spiritual diary after contact with nature, d) contemplative meditation with natural elements, e) symbolic ceremony, and f) outdoor spiritual reading.

e) *Arts*

The combination of nature and the arts is an effective tool for promoting physical, emotional, and cognitive health. It helps in rehabilitation of chronic diseases and supports mental health and general well-being.^103^ Its therapeutic objectives are: a) stimulating emotional expression and self-awareness, b) improving focus, mindfulness, and breathing, c) reducing stress levels, and d) promoting a sense of belonging, connection, and creativity.^104^, ^105^, ^106^, ^107^, ^108^

## Measuring the impact of nature prescription activities

The impact of nature prescription should be followed, evaluated and in the long term increasingly validated in various clinical study settings. The options for evaluation are listed in Table 4. The main clinical benefits having some objective evidence are summarized in Table 5.

**Table 4:** Evaluation options after the intervention with a nature prescription

Respiratory outcomes	a) Respiratory symptoms and disease control - ACT and cACT (asthma) or CAT (COPD) b) Rescue medication consumption (clinical diary) c) Lung function (spirometry or peak expiratory flow) d) Frequency of exacerbation (clinical diary)
Physiological biomarkers	a) Baseline heart rate (smartwatches or oximeter) b) Systemic blood pressure (validated measurement) c) Inflammatory markers d) Baecke questionnaire (physical activity)
Mental health and well-being (inflammation/stress)	a) Stress (EPS10) b) Anxiety and depression (HADS) c) Positive and negative affect scale (PANAS) d) Quality of life (SF36) e) Sleep (IGI) f) IL-6, IL-8, TNF-α; salivary/urinary cortisol
Program engagement and adherence	a) Session attendance (attendance list) b) Activity log (nature diary or app) c) Qualitative feedback (interviews or focus group)
Green exposure	a) Objective exposure (NDVI via GIS or geolocation) b) Perceived exposure (nature connection scale) c) Behavior change (before and after)
Biological outcomes (microbiota and inflammation)	Microbiota: Feces (gut) + skin/nasal/oropharyngeal swab
Suggested longitudinal assessment.	T0 = baseline (before starting); T1 = after 6 weeks; T2 = after 12 weeks; T3 = 3–6 months after completion (optional).
Satisfaction questionnaire (Appendix 1).

**Table 5:** Main clinical benefits of prescribing nature^5^^,^^6^^,^^8^^,^^109^^,^^110^

Synergistic physical and mental benefits	Improved lung-, muscle- and cardiovascular function, reduced stress, anxiety and systemic inflammation.
Improved treatment adherence	Increases adherence in chronic patients (particularly those who are sedentary or have low motivation).
Medication reduction	Moderate outdoor exercise can reduce the need for bronchodilators in patients with asthma and improve oxygenation in patients with mild to moderate COPD.
Social engagement	Promotes socialization and reduces isolation, especially among the elderly.
Improved weight loss	Psychological recovery and reduction of mental stress contribute to weight loss.

## Nature prescription program for children

Children will be subject to the same protocol, respecting their age and physical condition. All activities must be carried out under supervision, with attention to hydration, sun protection, breaks, and respiratory safety. They can be integrated into schools, health units, parks, and community areas.

**Activities include**:

• *Playful walks on trails or parks* with the goal of encouraging light movements with a focus on breathing. Activities will be guided by sensory exploration, natural treasure hunts, *shinrin-yoku*, or forest bathing, walking while observing trees, collecting leaves, and listening to the sounds of nature. These will last 20–30 min and will occur twice a week.
• *Breathing techniques activities* aim to train respiratory control. Slow inhalation and exhalation techniques will be used.
• *Exploration of blue spaces* through walks along shorelines (lake or beach) and playing with sand and water (without submersion), safe contact with lakes or beaches, drawing reflections in the water, and observing aquatic sounds and movements. The expected benefits include relaxation, stress reduction, and motoric stimulation.
• *Pediatric mindfulness* will be applied in the form of identifying nature sounds and consciously observing a leaf or flower, and through the game: “How do I feel here?"
• *Yoga for children* is performed with light positions with stories, simple poses with animal names (cat, snake, tree), group relaxation with music, focus on slow breathing and fun in 15–20-min sessions, accompanied by a trained instructor or educator.
• *Art and nature* activities will include outdoor drawing, painting with natural elements, creating mandalas with leaves and flowers, painting with clay or natural pigments, and sculpting with clay, promoting concentration, self-esteem, and ecological connection.

## Discussion

Nature prescription is based on a series of relatively well-documented scientific evidence that reinforces its institution.^12^^,^^109^ There is tentative evidence that contact with active nature reduces respiratory rate and effort, improves lung function and reduces airway inflammation. Exposure to biogenic chemicals like phytoncides has an immunomodulatory and anxiolytic effect, important for respiratory diseases.^111^

Outdoor activity stimulates deep nasal breathing, promoting better oxygenation. Regular light walking in natural environments improves lung function and exercise tolerance, reducing the need for bronchodilators. Spending time in green space helps reduce anxiety associated with dyspnea, common in COPD and asthma.

Nature prescription is safe, but we must consider some obstacles that can interfere with its implementation (bad weather, darkness, getting lost, wildlife, ticks, mosquitoes, among others). On the other hand, it is adaptable, as it can be prescribed individually (spending time in a park or green space nearby, contemplative observation, adapted yoga, or a short walk).

It is indicated for patients with mild to moderate symptoms, in places with low pollution, shade, and stable terrain. It is also low-cost, highly accessible, and well-received by patients. It also allows for the integration of complementary activities, as it can include mindfulness, meditation, yoga art, and spirituality, enhancing relaxation and treatment adherence.

Nature-based therapeutic prescription represents a low-cost, accessible, and clinically effective strategy, especially for patients with chronic respiratory diseases.^43^

The practices described are compatible with interdisciplinary approaches and can be gradually incorporated into patients' routines, if they are adapted to the clinical conditions, local context, and availability.^112^

Regarding the duration of the interventions, they ranged from 40 min to 2 h daily (also depending on the number of weekly sessions). The literature lacks consensus regarding the ideal length of exposure to nature. Studies suggest a shift in the paradigm from “*how long*” to “*how meaningful are the experiences*” in nature, emphasizing the importance of quality moments and the appreciation of emotional interactions with the natural environment to promote well-being, without focusing on establishing specific time frames or frequencies for these moments.^113^^,^^114^

Understanding nature as a health promoter signals a new paradigm in the field of preventive, psychosocial, and integrative medicine.

## Limitations

Most studies evaluating the impacts of green spaces on health do not use randomized clinical trials, but observational studies, making it difficult to establish causality. Few articles precisely quantify the percentage reduction in specific respiratory symptoms (eg, % reduction in asthma attacks) or a formal reduction in the consumption of respiratory relief medications, although there are positive associations.^7^^,^^110^

Overall, controlled interventions should be performed to better establish nature prescriptions in clinical practice. Furthermore, Evidence-based practice models for healthcare use are needed to assess available data for recommendations.^115^ There are several models, but none generally agreed. Thus, no specific one is followed in the present scoping review.

## Conclusion

Nature prescriptions offer relevant physiological and mental benefits for people with chronic respiratory diseases. Its application can significantly contribute to improving respiratory function, physical performance, quality of life, and overall well-being. There is an urgent need to reconcile the relationship between humans and nature—to work with nature, not against it.^116^

Healthcare professionals are in a key position to integrate public health promotion with environmental care. Their education is essential not only to treat symptoms but also to provide advice on lifestyle habits that promote patient health and protect our planet.^6^^,^^117^

The EAACI Guidelines and Recommendations on the Preventive Effects of Environmental Ecology and Green Spaces on Allergy and Asthma are certainly paving the way for improved awareness.^8^^,^^116^^,^^118^ Therefore, we recommend investing in professional training, clinical protocols, and public policies to enable evidence-based, safe and accessible use of nature in the management of chronic respiratory conditions and promotion of public health.

## Availability of data and materials

There are no data and materials collected.

## Author contributions

1 - Conceptualization; 2 - Writing; 3 - Review; 4 - Collaboration in structuring the protocol; 5 - Conducting a pilot test of the protocol.

M. Urrutia-Pereira (1, 2), D. Sole (2, 3), T. Winders (3), V. Riquelme Martinez (4), R.K. Bueno (4), D.C.P. Pellegrini (4), J. R. Bueno (4), F. H. Derré Torres (5), C.G. Alberti (5), B. Simoneto Marques (5), B. Angelo de Moraes (5), A.C. Sevá (5) D. Barba Kaestner (5), H.J. Chong-Neto (3), T. Haahtela (2, 3).

## Ethics approval

That's a review manuscript. No ethics approval are needed.

## Authors'consent

All authors consent for publication in the WAO Journal.

## Declaration of No AI use

We confirm that no GenAI or other AI-assisted technologies were utilized in any stage of this research or manuscript preparation. Nothing to disclose.

## Declaration of competing interest

T. Haahtela reports a lecturing fee from ALK/Abello.

All the other authors declare no conflict of interest.
