# Supplementary material for: Nature prescription for patients with chronic respiratory diseases
Source: World Allergy Organ J. 2026 Feb 27;19(3):101351. doi: 10.1016/j.waojou.2026.101351 (PMC12966752; doi:10.1016/j.waojou.2026.101351)
Supplement: Multimedia component 1 [file mmc1.docx]

**APPENDIX 1. Nature Prescription – Project Satisfaction Scale**

**Instructions:** Please answer the questions below based on your experience during the project activities.

**Part 1 – Overall satisfaction**

1. How satisfied are you with the project as a whole?

( ) Very dissatisfied ( ) Dissatisfied ( ) Neutral ( ) Satisfied ( ) Very satisfied

2. Were the outdoor activities enjoyable and accessible?

( ) Strongly disagree ( ) Disagree ( ) Neutral ( ) Agree ( ) Strongly agree

**Part 2 – Respiratory perception**

3. Did you feel any improvement in your breathing after participating in the activities?

( ) No improvement ( ) Slight improvement ( ) Moderate improvement ( ) Great improvement ( ) Very great improvement

4. Did you feel a reduction in the use of rescue medications? ( ) No ( ) Yes, a little ( ) Yes, moderately ( ) Yes, quite a bit ( ) Yes, completely

**Part 3 – Well-being and emotions**

5. Did you notice an improvement in your mood during the project?

( ) Didn't notice ( ) Slight improvement ( ) Moderate improvement ( ) Significant improvement ( ) Much improvement

6. Did the activities help reduce your stress or anxiety?

( ) Didn't help ( ) Helped a little ( ) Helped reasonably ( ) Helped a lot ( ) Helped completely

**Part 4 – Lifestyle and habits**

7. Did you increase your physical activity with the project?

( ) No ( ) Yes, a little ( ) Yes, moderately ( ) Yes, quite a bit ( ) Yes, very much.

8. Was there a reduction in your consumption of alcohol, tobacco, or other substances?

( ) No, if yes, which?

( ) Slight reduction ( ) Moderate reduction. ( ) Significant reduction

( ) Total elimination

**Part 5 – Connection with nature**

9. Did participating in the project increase your desire to be more in touch with nature?

( ) Strongly disagree ( ) Disagree ( ) Neutral ( ) Agree ( ) Strongly agree

10. Did you feel calmer or more centered after the activities?

( ) Never ( ) Rarely ( ) Sometimes ( ) Often ( ) Always

**Part 6 – Final comments (optional)**

11. What did you like most about the project?

12. What could be improved?
